# Health impacts of e-cigarette and traditional tobacco use in Shanghai male railway workers: A population-based retrospective cohort study

**DOI:** 10.18332/tid/209146

**Published:** 2025-10-18

**Authors:** Lishun Xiao, Natasha M. Weah, Yuan Chen, Jensen G. Weedor, Wenhong Wang, Lin Jiang, Xiaona Cong, Yansu Chen

**Affiliations:** 1 School of Public Health, Xuzhou Medical University, Xuzhou, China; 2 Jiangsu Engineering Research Center of Biological Data Mining and Healthcare Transformation, Xuzhou Medical University, Xuzhou, China; 3 Shanghai Railway Disease Prevention and Control Institute of China, Railway Shanghai Bureau Group Co., Shanghai, China

**Keywords:** health indicators, electronic cigarettes, dual users, traditional tobacco, accumulated smoking years

## Abstract

**INTRODUCTION:**

The health implications of electronic cigarette (e-cigarette) use remain uncertain despite their increasing global prevalence. This study evaluates the health hazards of e-cigarettes on railway workers by comparing the differences in clinical and biochemical health indicators resulting from exposure to different smoking methods.

**METHODS:**

Using a retrospective cohort design, this study analyzed 7719 routine physical examinations and clinical health records from male railway workers in Shanghai (March 2022). Participants were stratified into four smoking subgroups: non-users, e-cigarette users, cigarette smokers, and dual users (concurrent e-cigarette and cigarette use). A multinomial logistic regression analysis evaluated the potential health impacts associated with each type of cigarette use, while a linear regression analyzed the impact of accumulated smoking years on these health indicators.

**RESULTS:**

E-cigarette use was associated with increased odds of elevated systolic blood pressure (AOR=1.12; 95% CI: 1.01–1.24; AOR=1.18; 95% CI: 1.06–1.31) and heart rate (AOR=1.18; 95% CI: 1.06–1.33) per 10-unit increase, as well as reduced urine pH (AOR=0.64; 95% CI: 0.52–0.80; AOR=0.70; 95% CI: 0.56–0.88) compared to non-users and cigarette smokers. Compared to cigarette use, e-cigarette use was associated with higher hemoglobin levels (AOR=1.22; 95% CI: 1.05–1.42) and increased aspartate aminotransferase levels for every 10-unit increment (AOR=1.23; 95% CI: 1.01–1.51). Furthermore, relative to non-users, e-cigarette users showed higher levels of white blood cells and carcinoembryonic antigen, with the largest effect sizes observed among e-cigarette users compared to other subgroups. In addition, the number of accumulated smoking years significantly impacted clinical and biochemical health indicators in both cigarette and e-cigarette users.

**CONCLUSIONS:**

E-cigarette use was associated with adverse alterations in several clinical and biochemical health indicators, some of which were comparable to or more pronounced than those observed in cigarette smokers. Public health policies are necessary to regulate their use, particularly in occupational settings.

## INTRODUCTION

Tobacco smoking continues to be a substantial global public health concern, with wide-ranging implications that go beyond the individual smoker^1^. Despite ongoing public health efforts, countries such as China continue to struggle with smoking and its associated health consequences. This has prompted initiatives like the Healthy China 2030 agenda, which aims to address these challenges through strengthened tobacco control measures^2^.

While efforts to reduce traditional tobacco use persist, the prevalence of electronic cigarette (e-cigarette) use, commonly known as vaping, has surged^3,4^. E-cigarettes, often perceived as a less harmful alternative to traditional tobacco, have gained popularity, particularly among young adults, driven by their appealing designs, flavors, affordability, and discreet usage. This growing trend has sparked concerns regarding long-term health effects, nicotine dependence, and overall safety^5-9^. Recent studies have documented significant increases in e-cigarette use among both adolescents and adults across various regions^10-14^. In the United States, for instance, the prevalence of e-cigarette use among middle school students rose by 2.1% between 2020 and 2021, with approximately 10% (2.8 million) of students reported as users in 2023^10,11^. Similarly, in China, adult e-cigarette use increased by 0.3% between 2015 and 2019, while adolescent use in Jiangsu Province grew by 3.74% from 2019 to 2021^12,13^. Despite the rapid rise in usage, the health impacts of e-cigarette exposure remain insufficiently studied, particularly in occupational environments such as among railway workers.

Previous studies also reveal that e-cigarette use among current smokers may support smoking cessation^6-8^; however, some studies argue that, as consumer products, they fail to promote long-term cessation or prevent relapse^15-18^. These inconsistencies, including limited resources on the clinical and biochemical effects of e-cigarette use compared to cigarette use and dual use (i.e. both use of e-cigarettes and cigarettes) in occupational populations, particularly among adults in China, highlight the need for further study into the safety and health consequences of e-cigarette exposure.

This study evaluated the health hazards of e-cigarettes on railway workers by comparing the differences in clinical and biochemical health indicators resulting from exposure to different smoking methods. Additionally, the analysis investigated how the number of years spent smoking has affected these clinical and biochemical health indicators within each smoking subgroup.

## METHODS

### Study design and ethical approval

This retrospective cohort study was conducted among railway workers in the China Railway Shanghai Bureau Group through the Shanghai Railway Center for Disease Control and Prevention, China Railway Shanghai Bureau Group Co., Ltd. All railway workers in this group were enrolled in the study, totaling 64090 individuals. Their demographic and lifestyle characteristics were collected through self-reported electronic questionnaires, while clinical and biochemical health indicators were obtained through routine physical examinations. Railway workers were categorized into non-users, e-cigarette users, cigarette smokers, and dual users based on their responses to questions regarding smoking habits. The study adhered to the Declaration of Helsinki and was approved by the Ethics Committee of Xuzhou Medical University (Approval number: XZHMU-2023626).

### Questionnaire survey and physical examination

The self-reported questionnaire addressed various aspects including: age, marital status (unmarried, married), education level (junior high school or below, high/vocational school, university/technical college, Master’s degree or above), working age, weekly working hours (18–78 hours), night work shifts per week (0, ≤1, 2–3, ≥4) and income per month (0–2999, 3000–4999, 5000–7999, 8000–10999, 11000–29999, ≥30000 RMB). The questionnaire regarding smoking habits was derived from the questions: ‘Do you smoke?’ (smoking refers to taking one or more cigarettes per day for more than a year), with four response options ‘yes’, ‘sometimes’, ‘never’, ‘have already quit’; if the first two options were selected, a subsequent question was asked, ‘Have you ever used traditional tobaccos or e-cigarettes?’ with three options ‘only traditional tobaccos’ (usually traditional tobaccos and took e-cigarettes less than twice a year on average), only ‘e-cigarettes’ (usually e-cigarettes and took two or less traditional tobaccos a year on average), both ‘e-cigarettes and traditional tobaccos’ (except for the above two situations). Answers to these questions defined the four subgroups used in the present analysis. The accumulated smoking years were also collected from the questionnaire. In addition, information regarding railway workers lifestyle habits were collected including: self-reported sleep quality (very bad, poor, good, very good), lack of energy last month (no, occasionally, sometimes, often have), alcohol consumption (never, occasionally, sometimes, quit), smokers among 5 best friends (0, 1, 2, 3, 4, 5 persons), cognition of smoking hazard (harmless, hard to say, slightly, relatively, very harmful), and self-reported health quality (poor, average, good, very good).

Lastly, clinical and biochemical health indicators were measured by a Grade-A tertiary hospital as part of the participants’ routine physical examinations, following standardized procedures. Body mass index (BMI, kg/m^2^) was obtained by dividing weight by the square of height. Cardiovascular indicators included systolic blood pressure (SBP), diastolic blood pressure (DBP), and heart rate (HR), all measured using an automated and calibrated digital sphygmomanometer while participants were in a seated and rested position. Hematological parameters were assessed via complete blood count (CBC) tests, using venous blood samples collected under sterile conditions. These included red blood cell count (RBC), hemoglobin concentration (HGB), platelet count, and white blood cell count (WBC). Metabolic indicators comprised fasting blood glucose (FBG, mmol/L), which was measured after an overnight fast using standardized diabetic testing protocols. Lipid profile variables included serum triglycerides (mmol/L), total cholesterol (μmol/L), high-density lipoprotein (HDL-C, mmol/L), and low-density lipoprotein (LDL-C, mmol/L). These were analyzed using enzymatic colorimetric assays in certified clinical laboratories. Liver function and carcinogenic indicators were assessed through blood chemistry analysis and urinalysis. These included alanine aminotransferase (ALT, U/L), aspartate aminotransferase (AST, U/L), γ-glutamyl transferase (GGT, U/L), total bilirubin (μmol/L), direct bilirubin (μmol/L), total protein (g/L), albumin (g/L), urea nitrogen (mmol/L), uric acid (μmol/L), and creatinine (μmol/L). Tumor markers, including alpha-fetoprotein (AFP, u/mL) and carcinoembryonic antigen (CEA, ng/mL), were measured using immunoassay methods. Urine pH was assessed via standard urinalysis.

### Participants and data

**Figure 1:**
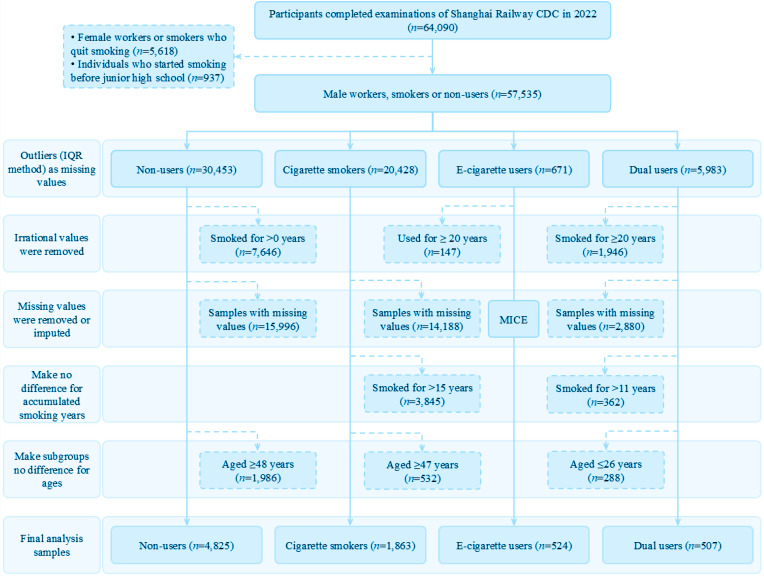
Flowchart illustrating the sample screening process for the study conducted among railway workers in Shanghai, China, March 2022 (N=7719)

In this study, all females and individuals who had quit smoking (n=5618) were excluded as the number of female smokers was very low (n=32), and smoking cessation could confound the effects of current cigarette exposure. Besides, we included female smokers in the screening process and found that only male smokers remained in the sample. Therefore, we excluded females in the first step. Outliers in continuous variables were identified using the interquartile range method and set as missing values. Variables with over 99% missing data (urinary glucose, urine protein, and urine ketones) were excluded. All remaining variables had <20% missing data. Individuals who started smoking before junior high school (when age minus accumulated smoking years was greater than 13 years, n=937) were eliminated as Chinese primary schools have strict smoking regulations for students, and it was unlikely for them to continue smoking during their primary school years. E-cigarettes were invented in China in 2003 and have been around for 20 years until 2022. Thus, e-cigarette users with accumulated smoking years ≥20 years were eliminated (n=147). Individuals with missing values were excluded from the non-user, cigarette smoker, and dual user subgroups. For e-cigarette users, Little’s test showed data were missing completely at random; therefore, missing values were imputed using multiple imputation by chained equations (MICE), following Rubin’s rules. To improve model interpretability, selected continuous clinical and biochemical indicators were discretized into ordinal categories using clinically meaningful bin widths. Most variables (e.g. blood pressure, heart rate, liver enzymes, hemoglobin, and creatinine) were binned into 10-unit intervals, while platelet count and uric acid were grouped into 100-unit intervals due to wider ranges. Each bin was labeled according to its upper bound, and numeric codes were assigned for regression modeling. All binned variables were checked and converted to a numeric format before analysis. After this rigorous data cleaning and imputation process, the final analytical sample consisted of 7719 participants: 4825 non-users, 1863 cigarette smokers, 524 e-cigarette users, and 507 dual users. The detailed flow for sample screening is shown in Figure 1 and the Supplementary file Section S1.

### Statistical analysis

All statistical analyses were done using R version 4.3.1 software. The continuous variables are described by the mean and standard deviation (SD), and the difference among the four subgroups was compared by analysis of variance (ANOVA) when homoscedasticity was satisfied; otherwise, the Kruskal-Wallis test was used. Categorical variables are summarized using frequencies (n) and percentages (%), and differences between subgroups are assessed using the chi-squared test (χ^2^). A multinomial logistic regression (MNLR) analysis was conducted to determine the potential health impacts associated with different types of cigarette use among male railway workers (see more details about MNLR in the Supplementary file Section S1). Sample size adequacy was determined according to the method of 10 events per variable (EPV) for a reliable regression analysis^19^. Given that 39 independent variables (including dummy variables) were included in the MNLR model, a minimum of 390 observations per outcome subgroup was required. The actual sample size in each subgroup exceeded this threshold, thus meeting the EPV criterion for model stability and validity. Before model fitting, multicollinearity was detected by running the corresponding linear regression model and calculating the variance inflation factor (VIF) for all independent variables included in the MNLR^20^. As shown in Supplementary file Table S1, all VIF values were below 10, indicating the absence of significant multicollinearity. The findings of the MNLR are presented in the form of adjusted odds ratios (AORs) together with their 95% confidence intervals (95% CIs). Akaike information criterion (AIC) and receiver operating characteristic (ROC) curve were used to determine how well the model classified different types of smokers correctly. Additional analysis assessed the effects of accumulated smoking years on clinical and biochemical health indicators through multiple linear regression analyses. Each health indicator was modeled as an independent variable. All statistical tests were two-tailed, and a p<0.05 was considered statistically significant.

## RESULTS

### Comparison of baseline characteristics

The study included 7719 male individuals (62.5% non-users, 24.1% cigarette smokers, 6.8% e-cigarette users, and 6.6% dual users) with an average age of 33.0 ± 7.0 years (Table 1). A significantly higher proportion of unmarried individuals was observed across all four subgroups compared to married individuals (p<0.001). Significant differences were also noted among the four subgroups in terms of education level (p<0.001), night work shifts (p=0.010), self-reported energy levels in the past month (p=0.013), and self-rated health quality (p<0.001). Specifically, education level varied significantly among non-users, cigarette smokers, e-cigarette users, and dual users. Differences in night work shifts were evident between non-users and dual users, while reports of reduced energy in the past month were more frequent among e-cigarette users and dual users. Lastly, self-rated health quality differed significantly across all four subgroups.

**Table 1:** Comparison of baseline demographic and lifestyle characteristics by smoking habits among male railway workers in Shanghai, China, March 2022 (N=7719)

*Characteristics*	*Overall* *n (%)*	*Non-users* *n (%)*	*Cigarette* *smokers* *n (%)*	*E-cigarette* *users* *n (%)*	*Dual users* *n (%)*	p*
**Total**, n	7719 (100)	4825 (62.5)	1863 (24.1)	524 (6.8)	507 (6.6)	
**Age** (years), mean ± SD	33.0 ± 7.0	33.0 ± 7.0	33.0 ± 6.0	32.0 ± 10.0	32.0 ± 7.0	0.532
**BMI** (kg/m^2^), mean ± SD	24.7 ± 3.2	24.5 ± 3.2	24.9 ± 3.3^a^	24.9 ± 3.4	25.0 ± 3.3^a^	**<0.001**
**Marital status**						**<0.001**
Unmarried	4307 (56.0)	2702 (56.0)	970 (52.0)	320 (61.0)	315 (62.0)	
Married	3412 (44.0)	2123 (44.0)	893 (48.0)	204 (39.0)	192 (38.0)	
**Education level**						**<0.001**
Junior high school or lower	78 (1.0)	33 (0.7)	10 (0.5)	29 (5.5)^a,b^	6 (1.2)^c^	
High/vocational school	1180 (15.0)	694 (14.0)	309 (17.0)	103 (20.0)	74 (15.0)	
University/technical college	6336 (82.0)	4004 (83.0)	1519 (82.0)	389 (74.0)	424 (84.0)	
Master’s degree or higher	125 (1.6)	94 (1.9)	25 (1.3)	3 (0.6)	3 (0.6)	
**Working status**						
Working age, mean ± SD	12.0 ± 9.0	12.0 ± 9.0	12.0 ± 8.0	11.0 ± 10.0	11.0 ± 8.0	0.309
Weekly working hours, mean ± SD	48.0 ± 11.0	47.0 ± 10.0	48.0 ± 11.0	47.0 ± 12.0	48.0 ± 11.0	0.077
**Night work shifts per week**						**0.010**
0 (day shifts only)	2063 (27.0)	1349 (28.0)	477 (26.0)	127 (24.0)	110 (22.0)^a^	
≤1	673 (8.7)	404 (8.4)	174 (9.3)	56 (11.0)	39 (7.7)	
2–3	3083 (40.0)	1928 (40.0)	733 (39.0)	199 (38.0)	223 (44.0)	
≥4	1900 (25.0)	1144 (24.0)	479 (26.0)	142 (27.0)	135 (27.0)	
**Income per month** (RMB)						0.203
0–2999	775 (10.0)	463 (9.6)	208 (11.0)	58 (11.0)	46 (9.1)	
3000–4999	1583 (21.0)	1053 (22.0)	338 (18.0)	103 (20.0)	89 (18.0)	
5000–7999	1716 (22.0)	1078 (22.0)	395 (21.0)	126 (24.0)	117 (23.0)	
8000–10999	1841 (24.0)	1105 (23.0)	486 (26.0)	120 (23.0)	130 (26.0)	
11000–29000	1610 (21.0)	995 (21.0)	405 (22.0)	103 (20.0)	107 (21.0)	
≥30000	194 (2.5)	131 (2.7)	31 (1.7)	14 (2.7)	18 (3.6)	
**Self-rated sleep quality**						0.112
Very bad	404 (5.2)	217 (4.5)	101 (5.4)	50 (9.5)	36 (7.1)	
Poor	2330 (30.0)	1470 (30.0)	554 (30.0)	129 (25.0)	177 (35.0)	
Good	3657 (47.0)	2328 (48.0)	896 (48.0)	231 (44.0)	202 (40.0)	
Very good	1328 (17.0)	810 (17.0)	312 (17.0)	114 (22.0)	92 (18.0)	
**Lack of energy last month**						**0.013**
No	2212 (29.0)	1399 (29.0)	493 (26.0)	178 (34.0)	142 (28.0) c	
Occasionally	3190 (41.0)	2007 (42.0)	803 (43.0)	195 (37.0)	185 (36.0)	
Sometimes	1144 (15.0)	704 (15.0)	294 (16.0)	74 (14.0)	72 (14.0)	
Often	1173 (15.0)	715 (15.0)	273 (15.0)	77 (15.0)	108 (21.0)	
**Alcohol consumption**						**<0.001**
Never	3457 (45.0)	2566 (53.0)	527 (28.0)	204 (39.0)	160 (32.0)	
Occasionally	3122 (40.0)	1830 (38.0)	901 (48.0)	170 (32.0)	221 (44.0)	
Regularly	1071 (14.0)	395 (8.2)	415 (22.0)	140 (27.0)	121 (24.0)	
Quit	69 (0.9)	34 (0.7)	20 (1.1)	10 (1.9)	5 (1.0)	
**Accumulated smoking years,** mean ± SD	2.2 ± 4.3	-	6.0 ± 5.3	5.3 ± 5.9	5.8 ± 4.0	0.070
**Smokers among 5 best friends**						**<0.001**
0	609 (7.9)	498 (10.0)	56 (3.0)^a^	39 (7.4)^a^	16 (3.2)^a,b,c^	
1	1030 (13.3)	793 (16.0)	157 (8.4)	54 (10.0)	26 (5.1)	
2	2250 (29.1)	1469 (30.0)	548 (29.0)	129 (25.0)	104 (21.0)	
3	2043 (26.5)	1173 (24.0)	584 (31.0)	130 (25.0)	156 (31.0)	
4	866 (11.2)	436 (9.0)	286 (15.0)	64 (12.0)	80 (16.0)	
5	921 (11.9)	456 (9.5)	232 (12.0)	108 (21.0)	125 (25.0)	
**Cognition of smoking hazard**						**<0.001**
Harmless	120 (1.6)	62 (1.3)	25 (1.3)^a^	24 (4.6)^a,b^	9 (1.8)^a,c^	
Hard to say	1140 (15.0)	505 (10.0)	363 (19.0)	164 (31.0)	108 (21.0)	
Slightly harmful	590 (7.6)	238 (4.9)	231 (12.0)	77 (15.0)	44 (8.7)	
Relatively harmful	2616 (34.0)	1455 (30.0)	793 (43.0)	157 (30.0)	211 (42.0)	
Very harmful	3253 (42.0)	2565 (53.0)	451 (24.0)	102 (19.0)	135 (27.0)	
**Self-rated health quality**						**<0.001**
Poor	638 (8.3)	392 (8.1)	144 (7.7)	40 (7.6)^a,b^	62 (12.0)^c^	
Average	3217 (42.0)	1987 (41.0)	843 (45.0)	179 (34.0)	208 (41.0)	
Good	2069 (27.0)	1332 (28.0)	468 (25.0)	145 (28.0)	124 (24.0)	
Very good	1795 (23.0)	1114 (23.0)	408 (22.0)	160 (31.0)	113 (22.0)	

Chi-squared test used for subgroup comparison of categorical variables. Kruskal-Wallis test used for subgroup comparison of continuous or ordinal variables.

The superscript symbols a, b, and c represent a statistically significant difference compared to the subgroups of non-users, cigarette smokers, and e-cigarette users, respectively, by Wilcoxon test. RMB: 1000 Chinese Renminbi about US$140.

* In bold p<0.05, statistically significant.

For non-users, individuals with 2 smokers among their 5 best friends and those who had never consumed alcohol had the highest proportion, while for cigarette smokers, those with 3 smokers among their 5 best friends who occasionally consumed alcohol had the highest proportion. Perception of smoking hazards also differed: over 50% of non-users recognized smoking as very harmful, while only 40% of cigarette smokers and dual users considered it relatively harmful, and over 30% of e-cigarette users found the risk uncertain (‘hard to say’) (Table 1).

Regarding clinical and biochemical health indicators, e-cigarette users had the highest SBP and DBP, HR, HGB, ALT, AST, and CEA, while albumin, AFP, and urine pH were the lowest. Non-users had the lowest values of triglyceride, WBC, platelet, and GGT, but the highest values of HDL-C and total protein. Cigarette smokers had the lowest values of total bilirubin and direct bilirubin, but no difference was observed between non-users and e-cigarette users. Although platelet levels varied significantly among subgroups (p=0.02), pairwise comparisons were not statistically significant, likely due to sample variability or the conservative nature of Tamhane’s test. Further subgroup characteristics are detailed in Table 2.

**Table 2:** Comparison of baseline clinical characteristics by smoking status among male railway workers in Shanghai, China, March 2022 (N=7719)

*Characteristics*	*Overall* *Mean ± SD*	*Non-users* *Mean ± SD*	*Cigarette* *smokers* *Mean ± SD*	*E-cigarette* *users* *Mean ± SD*	*Dual users* *Mean ± SD*	p*
Total, n	7719 (100)	4825 (62.5)	1863 (24.1)	524 (6.8)	507 (6.6)	
Systolic blood pressure (mmHg)	124 ± 13.00	124 ± 13.00	123 ± 13.00	125 ± 14.00^a,b^	122 ± 13.00^a,c^	**<0.001**
Diastolic blood pressure (mmHg)	75 ± 10.00	76 ± 10.00	75 ± 10.00	77 ± 11.00^b^	74 ± 10.00^a,c^	**<0.001**
Heart rate (beats/min)	73 ± 9.00	73 ± 9.00	72 ± 9.00	75 ± 10.00^a,b^	72 ± 8.00^a,c^	**<0.001**
Fasting blood glucose (mmol/L)	5.14 ± 0.53	5.15 ± 0.53	5.12 ± 0.52	5.13 ± 0.58	5.13 ± 0.55	0.503
Triglyceride (mmol/L)	1.47 ± 0.73	1.42 ± 0.70	1.55 ± 0.76^a^	1.52 ± 0.82^a^	1.52 ± 0.72^a^	**<0.001**
Total cholesterol (μmol/L)	4.70 ± 0.79	4.70 ± 0.79	4.70 ± 0.77	4.67 ± 0.86	4.72 ± 0.79	0.790
High-density lipoprotein (mmol/L)	1.25 ± 0.24	1.27 ± 0.24	1.24 ± 0.24^a^	1.23 ± 0.25^a^	1.23 ± 0.24^a^	**<0.001**
Low-density lipoprotein (mmol/L)	2.82 ± 0.66	2.82 ± 0.66	2.82 ± 0.64	2.78 ± 0.73	2.83 ± 0.65	0.621
Hemoglobin (g/L)	155 ± 9.00	155 ± 9.00	155 ± 9.00	157 ± 9.00^a,b^	155 ± 9.00^c^	**<0.001**
Red blood cell (1012/L)	5.14 ± 0.33	5.14 ± 0.33	5.13 ± 0.33	5.17 ± 0.35	5.1 ± 0.32^c^	**0.007**
White blood cell (109/L)	6.34 ± 1.37	6.19 ± 1.32	6.56 ± 1.43^a^	6.74 ± 1.52^a^	6.50 ± 1.31^a,c^	**<0.001**
Platelet (/mcL)	233 ± 47.00	231 ± 47.00	234 ± 48.00	235 ± 49.00	235 ± 47.00	**0.020**
Alanine aminotransferase (U/L)	26 ± 12.00	25 ± 12.00	26 ± 12.00	27 ± 14.00^a^	26 ± 12.00	**0.001**
Aspartate aminotransferase (U/L)	20.60 ± 5.10	20.50 ± 5.00	20.6 ± 5.30	21.4 ± 5.90^a,b^	20.60 ± 5.10	**0.002**
γ-glutamyl transferase (U/L)	28 ± 14.00	27 ± 14.00	30 ± 15.00^a^	31 ± 17.00^a^	30 ± 15.00^a^	**<0.001**
Total bilirubin (μmol/L)	14.30 ± 4.80	14.60 ± 4.70	13.80 ± 4.70^a^	14.10 ± 5.00	13.90 ± 4.70^a^	**<0.001**
Direct bilirubin (μmol/L)	4.14 ± 1.57	4.21 ± 1.58	3.99 ± 1.51^a^	4.14 ± 1.67	4.08 ± 1.55	**<0.001**
Total protein (g/L)	74.4 ± 3.8	74.70 ± 3.80	74.00 ± 3.70^a^	73.90 ± 3.90^a^	73.80 ± 3.70^a^	**<0.001**
Albumin (g/L)	47.13 ± 2.48	47.19 ± 2.49	47.11 ± 2.39	46.82 ± 2.67^a^	46.93 ± 2.48	**0.003**
Urea nitrogen (mmol/L)	5.06 ± 1.08	5.07 ± 1.07	5.08 ± 1.09	4.98 ± 1.10	5.05 ± 1.13	0.272
Uric acid (μmol/L)	381 ± 74.00	380 ± 74.00	381 ± 72.00	385 ± 76.00	383 ± 74.00	0.407
Creatinine (μmol/L)	76 ± 11.00	76 ± 11.00	76 ± 10.00	75 ± 11.00	76 ± 11.00	0.105
Alpha-fetoprotein (u/mL)	2.66 ± 1.50	2.65 ± 1.52	2.72 ± 1.51	2.52 ± 1.30^b^	2.74 ± 1.48	**0.031**
Carcinoembryonic antigen (ng/mL)	1.77 ± 0.99	1.72 ± 0.98	1.84 ± 0.97^a^	1.90 ± 1.05^a^	1.82 ± 0.98	**<0.001**
Urine pH	5.92 ± 0.42	5.94 ± 0.42	5.91 ± 0.41	5.83 ± 0.47^a,b^	5.94 ± 0.40^c^	**<0.001**

ANOVA, Kruskal-Wallis test used for subgroup comparison of continuous or ordinal variables.

The superscript symbols a, b, and c represent a statistically significant difference compared to the subgroups of non-users, cigarette smokers, and e-cigarette users, respectively, by Wilcoxon test.

* In bold p<0.05, statistically significant.

### Effects of different cigarette exposure

**Figure 2:**
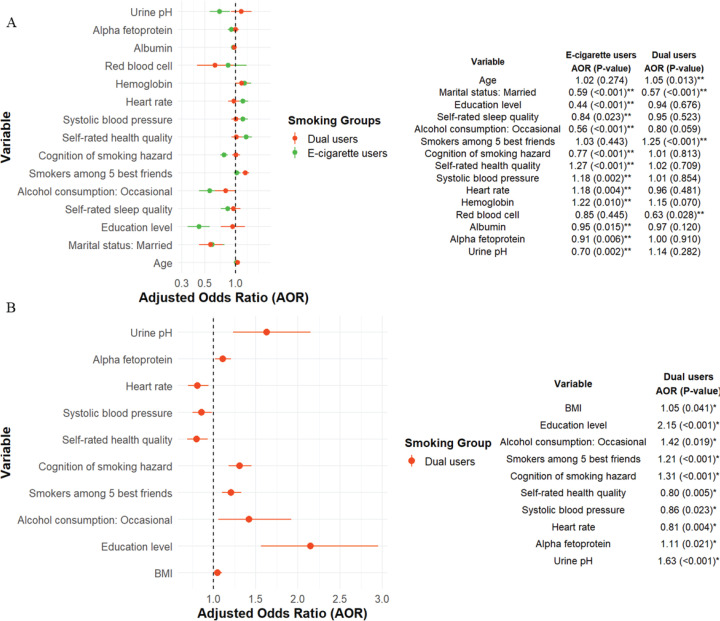
Forest plots comparing the effects of e-cigarette exposure versus other product exposures on a range of clinical and biochemical health indicators among male railway workers in Shanghai, China, March 2022 (N=7719): A) Adjusted odds ratios and 95% confidence intervals (CIs) for various health indicators when comparing e-cigarette users with cigarette smokers; B) Comparison between dual users (individuals who use both e-cigarettes and traditional cigarettes) and e-cigarette users. Each forest plot illustrates the direction and strength of associations between different types of product exposure and the selected biochemical health indicators, with models adjusted for relevant covariates

All MNLR models were adjusted for potential confounders, including age, body mass index, marital status, education level, monthly income, night work shifts, self-rated sleep quality, and alcohol consumption. The results of the MNLR, using non-users as the reference group, are presented in Table 3, and the corresponding results with cigarette smokers and e-cigarette users as references were demonstrated in Figure 2 and Supplementary file Table S2 to facilitate comparison across all subgroups.

**Table 3:** Multinomial logistic regression analysis for smoking status among male railway workers in Shanghai, China, March 2022 (N=7719) (reference category: non-users)

*Variables*	*Cigarette smokers*		*E-cigarette users*		*Dual users*	
	*AOR (95% CI)*	*p*	*AOR (95% CI)*	*p*	*AOR (95% CI)*	p*
Age (years)	0.99 (0.97–1.01)	0.323	1.01 (0.97–1.04)	0.634	1.04 (1.00–1.08)	**0.050**
Marital status: Married	1.20 (1.02–1.40)	**0.024**	0.71 (0.54–0.93)	**0.014**	0.69 (0.53–0.90)	**0.006**
Education level	0.86 (0.74–1.01)	0.062	0.38 (0.30–0.48)	**<0.001**	0.81 (0.63–1.05)	0.118
Working age	0.99 (0.98–1.01)	0.600	0.97 (0.94–0.99)	**0.026**	0.96 (0.93–1.00)	**0.032**
Income per month	1.02 (0.98–1.07)	0.350	1.04 (0.97–1.12)	0.257	1.07 (1.00–1.15)	**0.056**
Self-rated sleep quality	1.09 (1.00–1.20)	**0.058**	0.92 (0.79–1.06)	0.244	1.04 (0.90–1.20)	0.599
Alcohol consumption occasionally	2.41 (2.11–2.75)	**<0.001**	1.35 (1.07–1.69)	**0.010**	1.92 (1.54–2.40)	**<0.001**
Alcohol consumption regularly	4.55 (3.80–5.46)	**<0.001**	4.41 (3.37–5.77)	**<0.001**	4.06 (3.06–5.37)	**<0.001**
Alcohol consumption quit	3.11 (1.74–5.57)	**<0.001**	4.85 (2.24–10.50)	**<0.001**	2.39 (0.89–6.42)	0.083
Smokers among 5 best friends	1.26 (1.20–1.31)	**<0.001**	1.29 (1.20–1.39)	**<0.001**	1.57 (1.46–1.69)	**<0.001**
Cognition of smoking hazard	0.64 (0.61–0.68)	**<0.001**	0.50 (0.46–0.54)	**<0.001**	0.65 (0.60–0.70)	**<0.001**
Self-rated health quality	0.98 (0.91–1.05)	0.523	1.24 (1.11–1.40)	**<0.001**	1.00 (0.89–1.13)	0.994
Systolic blood pressure	0.95 (0.89–1.01)	0.111	1.12 (1.01–1.24)	**0.029**	0.96 (0.87–1.06)	0.436
Diastolic blood pressure	0.93 (0.86–1.01)	**0.084**	0.91 (0.80–1.04)	0.172	0.84 (0.74–0.97)	**0.015**
Heart rate	0.93 (0.87–0.99)	**0.029**	1.10 (0.98–1.23)	0.096	0.93 (0.77–1.13)	**0.041**
High-density lipoprotein	0.71 (0.52–0.97)	**0.031**	0.54 (0.32–0.90)	**0.019**	0.48 (0.29–0.81)	**0.006**
Low-density lipoprotein	0.86 (0.71–1.04)	0.117	0.69 (0.51–0.93)	**0.014**	0.81 (0.60–1.11)	0.188
Hemoglobin	1.22 (1.12–1.34)	**<0.001**	1.50 (1.29–1.73)	**<0.001**	1.41 (1.22–1.63)	**<0.001**
Red blood cell	0.55 (0.43–0.70)	**<0.001**	0.47 (0.31–0.69)	**<0.001**	0.35 (0.23–0.51)	**<0.001**
White blood cell	1.21 (1.16–1.27)	**<0.001**	1.27 (1.18–1.37)	**<0.001**	1.16 (1.07–1.25)	**<0.001**
Aspartate aminotransferase	1.03 (0.91–1.17)	0.597	1.27 (1.04–1.55)	**0.020**	1.01 (0.82–1.24)	0.937
γ-glutamyl transferase	1.05 (1.00–1.11)	**0.035**	1.06 (0.98–1.14)	0.142	1.08 (1.00–1.16)	0.062
Total bilirubin	0.98 (0.96–0.99)	**0.013**	0.98 (0.95–1.01)	0.242	0.97 (0.95–1.00)	0.075
Total protein	0.94 (0.92–0.96)	**<0.001**	0.94 (0.91–0.97)	**<0.001**	0.94 (0.92–0.97)	**<0.001**
Albumin	1.05 (1.02–1.07)	**0.001**	0.99 (0.95–1.03)	0.604	1.01 (0.97–1.05)	0.696
Alpha-fetoprotein	1.03 (0.99–1.07)	0.146	0.93 (0.87–0.99)	**0.046**	1.03 (0.97–1.10)	0.316
Carcinoembryonic antigen	1.12 (1.06–1.19)	**<0.001**	1.20 (1.09–1.33)	**<0.001**	1.13 (1.02–1.24)	**0.016**
Urine pH	0.92 (0.80–1.06)	0.240	0.64 (0.52–0.80)	**<0.001**	1.04 (0.84–1.31)	0.702

AOR: adjusted odds ratio; adjusted for age, body mass index, education level, monthly income, alcohol consumption, self-rated sleep quality, work shift, and marital status.

* In bold p<0.05, statistically significant.

The findings indicated that age was positively associated with dual users compared to both non-users and cigarette smokers (AOR=1.04; 95% CI: 1.00–1.08; AOR=1.05; 95% CI: 1.01–1.10) among male railway personnel. Being married was significantly and positively associated with cigarette smoking (AOR=1.20; 95% CI: 1.02–1.40) compared to non-use, while it was negatively associated with e-cigarette use and dual use compared to both non-use (AOR=0.71; 95% CI: 0.54–0.93; AOR=0.69; 95% CI: 0.53–0.90) and cigarette smoking (AOR=0.59; 95% CI: 0.44–0.78; AOR=0.57; 95% CI: 0.43–0.75). Participants who reported a higher frequency of alcohol consumption were more likely to be users of cigarettes, e-cigarettes, or dual cigarettes compared to non-users, even among those who indicated that they had quit drinking alcohol. Individuals who recognized smoking as a hazard exhibited diminished odds of being cigarette smokers, e-cigarette users, and dual users (AOR=0.64; 95% CI: 0.61–0.68; AOR=0.50; 95% CI: 0.46–0.54; AOR=0.65; 95% CI: 0.60–0.70) relative to non-users; it also holds for e-cigarette users relative to cigarette smokers (AOR=0.77; 95% CI: 0.71–0.84). Conversely, individuals who recognized smoking as a hazard exhibited elevated odds of being dual users relative to e-cigarette users (AOR=1.31; 95% CI: 1.18–1.45). E-cigarette users reported a higher self-rated health quality compared to non-users and cigarette smokers (AOR=1.24; 95% CI: 1.11–1.40; AOR=1.27; 95% CI: 1.13–1.44).

Compared to non-users, results from the MNLR indicated that cigarette smokers, e-cigarette users, and dual users had increased odds of elevated hemoglobin levels, WBC counts, and CEA levels. Specifically, for every 10 g/L increase in hemoglobin, the odds of use were significantly higher among cigarette smokers (AOR=1.22; 95% CI: 1.12–1.34), e-cigarette users (AOR=1.50; 95% CI: 1.29–1.73), and dual users (AOR=1.41; 95% CI: 1.22–1.63). Similarly, the odds of elevated WBC counts were higher among cigarette smokers (AOR=1.21; 95% CI: 1.16–1.27), e-cigarette users (AOR=1.27; 95% CI: 1.18–1.37), and dual users (AOR=1.16; 95% CI: 1.07–1.25). For CEA levels, the odds were also significantly increased per one-unit rise among cigarette smokers (AOR=1.12; 95% CI: 1.06–1.19), e-cigarette users (AOR=1.20; 95% CI: 1.09–1.33), and dual users (AOR=1.13; 95% CI: 1.02–1.24). Relative to cigarette smokers, e-cigarette users exhibited increased odds of elevated hemoglobin levels (AOR=1.22; 95% CI: 1.05–1.42) per 10 g/L increase.

Compared to cigarette smokers, e-cigarette users showed significantly increased odds of elevated heart rate (AOR=1.18; 95% CI: 1.06–1.33) per 10 beats per minute increase (Figure 2). Additionally, e-cigarette users demonstrated increased odds of elevated SBP (AOR=1.12; 95% CI: 1.01–1.24 vs non-users; AOR=1.18; 95% CI: 1.06–1.31 vs cigarette smokers) and elevated AST levels (AOR=1.27; 95% CI: 1.04–1.55 vs non-users; AOR=1.23; 95% CI: 1.00–1.51 vs cigarette smokers), per 10-unit increase. E-cigarette users exhibited reduced odds of higher urine pH levels (AOR=0.64; 95% CI: 0.52–0.80 vs non-users; AOR=0.70; 95% CI: 0.56–0.88 vs cigarette smokers), while dual users showed increased odds of higher urine pH levels compared to e-cigarette users (AOR=1.63; 95% CI: 1.23–2.15).

The ROC curve for MNLR is presented in Supplementary file Figure S1. The model showed varying levels of discrimination between non-users and the three smoking subgroups. Specifically, the model’s ability to differentiate between non-users and e-cigarette users was the highest (area under curve, AUC=0.794), followed by non-users and dual users (AUC=0.777), and non-users and cigarette smokers (AUC=0.752). These AUC scores were all greater than 70%, suggesting acceptable discrimination, and the corresponding results were reliable^21^. The corresponding pseudo coefficient of determination is 0.26, representing that 26.0% of the total variation was explained by the model.

### The effects of accumulated smoking years

**Figure 3:**
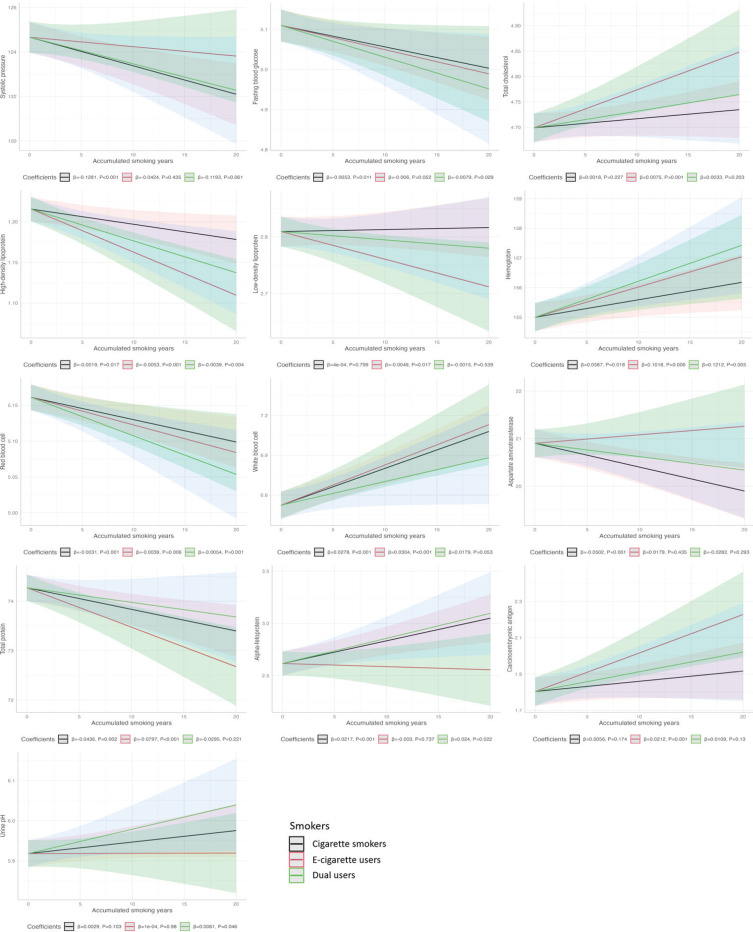
Unadjusted estimates from a linear regression model assessing the effect of accumulated smoking years on selected clinical health indicators across the three smoking subgroups (e-cigarette users, cigarette smokers, and dual users) of male railway workers in Shanghai, China, March 2022 (N=2894)

The results of the multiple linear regression analysis evaluating the impact of accumulated smoking years on clinical and biochemical indicators are presented in Figure 3 and Supplementary file Table S3. For cigarette smokers, the accumulated smoking years exhibited a significant and negative effect on SBP and AST (β= -0.1281, p<0.001; β= -0.0502, p=0.001), while no significant effects were observed in the other two smoking subgroups. The accumulated smoking years had negative effects on fasting blood glucose in both cigarette smokers and dual users (β= -0.0053, p=0.011; β= -0.0079, p=0.029), while it holds conversely true for alpha-fetoprotein (β=0.0217, p<0.001; β=0.0240, p=0.022). For e-cigarette users, the accumulated smoking years exhibited a positive effect on total cholesterol and CEA (β=0.0075, p=0.001; β=0.0212, p=0.001) and a negative effect on low-density lipoprotein (β= -0.0049, p=0.017), while no significant effects were observed in the other two smoking subgroups. Both high-density lipoprotein (β= -0.0019, p=0.017; β= -0.0053, p<0.001; β= -0.0039, p=0.004) and red blood cell (β= -0.0031, p=0.001; β= -0.0039, p=0.006; β= -0.0054, p=0.001) were negatively affected by the accumulated smoking years in all the three smoking subgroups; while it holds conversely true for hemoglobin (β=0.0587, p=0.018; β=0.1018, p=0.006; β=0.1212, p=0.005). WBC was positively affected by the accumulated years in both cigarette smokers and e-cigarette users (β=0.0278, p<0.001; β=0.0304, p<0.001); while it holds conversely true for total protein (β= -0.0436, p=0.002; β= -0.0797, p<0.001). For dual users, the accumulated smoking years showed a positive impact on urine pH (β=0.0061, p=0.046).

## DISCUSSION

This study evaluated the health hazards of e-cigarettes on railway workers by comparing the differences in clinical and biochemical health indicators resulting from exposure to different smoking methods. The findings provide comparative insights into how each type of tobacco product use is associated with various indicators. Additionally, the analysis explored how accumulated smoking years influenced these clinical and biochemical health indicators within each smoking subgroup.

Firstly, certain demographic and lifestyle factors may influence smoking behaviors among male railway workers. For instance, older participants were more likely to be dual users of cigarettes and e-cigarettes, whereas individuals with longer working durations were less likely to use e-cigarettes or engage in dual behaviors. However, univariate analysis revealed no significant differences in mean age or working years across the three smoking subgroups, suggesting that these factors were not key determinants of smoking behavior in this population. In addition, married individuals who recognized smoking as a hazard were less likely to be e-cigarette users, while individuals with higher alcohol consumption frequency were more inclined to smoke cigarettes rather than e-cigarettes. This preference may be attributed to the social practice of exchanging traditional tobacco as a gesture of goodwill in social settings, a function that e-cigarettes do not fulfill. Also, those who use e-cigarettes may perceive them as less harmful, as e-cigarette users reported higher self-rated health quality compared to other subgroups, whereas dual users tended to evaluate their health quality more negatively than e-cigarette users.

Secondly, the three types of smoking behavior demonstrated varying effects on these indicators. Although e-cigarettes do not contain the approximately 7000 chemicals found in traditional cigarettes, they still pose significant health risks due to the constituents of e-liquids^22,23^. Our findings revealed that e-cigarette use, in comparison to non-use, cigarette smoking, and dual use, was associated with elevated systolic blood pressure and heart rate. These effects may be attributed to the chemical components in e-liquids, which include nicotine, aldehydes, and other additives. The observed elevation in cardiovascular parameters among e-cigarette users suggests an increased risk of cardiac complications, even with relatively modest exposure levels^24^.

Traditional tobacco smoking is well-established in literature to increase hemoglobin levels through mechanisms involving chronic hypoxia^25,26^. Consistent with this, our study found elevated hemoglobin levels among cigarette smokers. However, a more striking observation was that e-cigarette use, compared to both non-use and cigarette smoking, was associated with a significantly greater increase in hemoglobin levels. One possible explanation could be hypoxia-induced erythropoiesis resulting from exposure to constituents in e-cigarette aerosols, such as propylene glycol and vegetable glycerin, which may impair pulmonary function and reduce oxygen exchange over time. Additionally, the presence of ultrafine particles and heavy metals in e-cigarette vapor may induce systemic inflammation or oxidative stress, thereby indirectly stimulating erythropoiesis. This finding is particularly concerning, as elevated hemoglobin concentrations have been linked to increased blood viscosity, higher risk of thrombosis, and adverse cardiovascular outcomes, including stroke^27,28^. The stronger association observed in e-cigarette users underscores the potential for greater cardiovascular harm in this subgroup and highlights the urgent need for further research on the long-term health consequences of e-cigarette exposure.

The current study also found that e-cigarette use, compared to non-use, was linked to increased WBC levels and CEA, with e-cigarette users showing the highest odds ratio among the three smoking subgroups. Notably, the effect sizes for WBC and CEA were greater among e-cigarette users than cigarette smokers. This study is among the first to report a significant association between e-cigarette use and elevated CEA levels. Acute inhalation of e-cigarette aerosols may induce oxidative stress and inflammatory responses due to the presence of harmful chemicals such as nicotine, formaldehyde, and acrolein^29^ which can stimulate immune activity and elevate WBC counts^30^. Furthermore, toxic components within e-cigarette vapor may cause cellular damage^31,32^, potentially contributing to increased CEA levels, a biomarker often associated with carcinogenic processes.

Additionally, our study revealed that e-cigarette users had a higher risk of elevated AST levels compared to non-users and cigarette smokers. E-cigarettes produce reactive oxygen species that increase oxidative stress, potentially causing liver cell damage^32,33^. While alcohol consumption and traditional smoking are known to elevate AST levels, caution is warranted when interpreting the effects of e-cigarettes. Although often perceived as a safer alternative, more frequent use of e-cigarettes may inadvertently increase exposure to harmful substances, thereby contributing to liver injury. These findings further highlight the need for long-term investigations.

Finally, the accumulated smoking years had a significant effect on clinical and biochemical health indicators among cigarette smokers and e-cigarette users; however, the effect was greater among e-cigarette users, including high-density lipoprotein, low-density lipoprotein, hemoglobin, red blood cell, WBC, and total protein. The dual users showed the highest effect values of accumulated smoking years on fasting blood glucose, hemoglobin, red blood cell, and alpha-fetoprotein. These findings emphasize the need for further study into the long-term health implications of smoking duration, particularly in occupational populations.

### Limitations

Several limitations in this study should be acknowledged. First, the retrospective cohort design may introduce recall bias, particularly in self-reported variables such as cumulative smoking duration, which may differ from actual smoking histories. Second, the study did not differentiate between specific e-cigarette device types (e.g. vapor-based vs pod-based systems), limiting our ability to evaluate the potentially varied health impacts of different product designs. Third, the study sample consisted exclusively of male railway workers in Shanghai, China, and excluded females and certain smoking subgroups. This occupational and gender-specific sampling may introduce selection bias and limit the generalizability of the findings to broader or more diverse populations. Fourth, although alcohol intake was adjusted for in the analysis, AST levels, which were found to be elevated among e-cigarette users, may also be influenced by alcohol consumption. Given that alcohol use was self-reported and detailed drinking patterns were not captured, the potential for residual confounding remains. Fifth, clinical and biochemical health indicators were obtained during routine health examinations, which may be affected by unmeasured lifestyle factors such as diet, physical activity, or occupational exposures, further limiting external validity. Lastly, the findings are based on data from the Shanghai Railway Bureau, which may not reflect the broader working population or international contexts. Future studies should employ longitudinal designs, include female participants and non-occupational cohorts, and incorporate more detailed lifestyle and exposure assessments to validate and expand upon the observed associations.

## CONCLUSIONS

E-cigarette use among railway workers in the China Railway Shanghai Bureau Group was associated with adverse alterations in several clinical and biochemical health indicators, some of which were comparable to or more pronounced than those observed in cigarette smokers. Public health policies are necessary to regulate their use, particularly in occupational settings.

## Supplementary Material



## Data Availability

The data supporting this research are available from the authors on reasonable request. The source R codes and execution results of the present study can be downloaded from the following hyperlinks: https://gitee.com/xiaostat/data-analysis-codes/tree/master/EffectsOfECigaretteExposure.
