# Supplementary material for: Health impacts of e-cigarette and traditional tobacco use in Shanghai male railway workers: A population-based retrospective cohort study
Source: Tob Induc Dis. 2025 Oct 18;23:10.18332/tid/209146. doi: 10.18332/tid/209146 (PMC12535226; doi:10.18332/tid/209146)
Supplement: Supplementary file 1 [file TID-23-158-s1.pdf]

Supplementary Materials for

Health Impacts of E-Cigarette and Traditional Tobacco Use in Shanghai Male Railway Workers: A Population-Based Retrospective Cohort Study.

Section S1 Materials and Methods

1.1 Participants and data

All females and individuals who had quit smoking ($n = 5,618$) were excluded as the number of female smokers was very low ($n = 32$), and smoking cessation could confound the effects of current cigarette exposure. Outliers in continuous variables were identified using the interquartile range method and set as missing values. Variables with over 99% missing data (urinary glucose, urine protein, and urine ketones) were excluded. All remaining variables had <20% missing data. Individuals who started smoking before junior high school (age minus accumulated smoking years was greater than 13 years, n=937) were eliminated as Chinese primary schools have strict smoking regulations for students, and it was unlikely for them to continue smoking during their primary school years. A total of 30,453 individuals were classified as non-users, 20,428 were classified as cigarette smokers, 671 were classified as e-cigarette users, and 5,983 as dual users. E-cigarettes were invented in China in 2003 and have been around for 20 years until 2022. Thus, e-cigarette users with accumulated smoking years greater than or equal to 20 years were eliminated (n=147). To ensure the accumulated smoking years were not different between dual users and e-cigarette users, dual users with accumulated smoking years greater than or equal to 20 were eliminated (n=1,946). Non-users with accumulated smoking years greater than 0 were eliminated (n=7,646). The individuals with missing values were eliminated for dual users, cigarette smokers, and non-users (n = 2,880, 14,188, 15,996, resp.). To address the missing values for e-cigarette users, we performed Little's test and found that the missing values were missing completely at random. Therefore, we imputed these missing values for e-cigarette users by multiple imputations through the chained equations (MICE) method, which followed Rubin's rule. To ensure that there existed no difference for accumulated smoking years among cigarette smokers, e-cigarette users, and dual users, cigarette smokers (dual users, resp.) with accumulated smoking years greater than 15 (greater than 11, resp.) were eliminated (n=3,845, 362, resp.). After that, no difference in accumulated smoking years was found among the three subgroups via Games-Howell's test. Similarly, to make sure that there existed no difference for ages, non-users (cigarette smokers, dual users, resp.) with ages ≥48 (≥47, ≤26, resp.) were eliminated (n=1,986, 532, 288, resp.). After that, there existed no difference in ages among the four subgroups via Games-Howell's test. Finally, the final sample size of 7,719 used in this analysis includes non-users (4,825), cigarette smokers (1,863), e-cigarette users (524), and dual users (507), respectively.

1.2 Multinomial logistic regression model concept

A multinomial logistic regression (MNLR) was conducted to determine the effects of exposure to different smoking types on male railway workers. Notice that the correlation results were equivalent when swapping the

position of the outcome variable and a certain explanatory variable. For instance, assume that $Y = \{1,2,3,4\}$ represents the four aforementioned subgroups, and X_j, $j = 1,2,\cdots,m$ the explanatory variables, from which a MNLR equation and some linear regression equations can be built as follows,

$$\text{logit}[P(Y = i|X)] = \ln\left[\frac{P(Y = i|X)}{P(Y = 1|X)}\right] = \beta_{i0} + \sum_{j=1}^{m} \beta_{ij}X_j + \varepsilon_1, \;\; i = 2,3,4,$$

$$X_l = \beta_{l0} + \sum_{j\neq l} \gamma_{lj}X_j + \sum_{i=2}^{4} \alpha_{li}Y_i + \varepsilon_2, \qquad l = 1,2,\cdots,m.$$

Then, if we want to check the effects on X_l for the exposure $Y = 2$ references to $Y = 1$, the coefficients α_{l2} and β_{2l} enjoy the same sign and hypothesis testing. Therefore, with the outcome variable being set as the four subgroups and other variables as explanatory variables, performing the MNLR operation only once can obtain the impacts of different cigarette-type exposures on all variables.

Table S1 Generalized Variance Inflictor Factor (GVIF) values for the multinomial logistic regression analysis

**Variables**	**GVIF**
Age	2.973726
BMI	1.253244
Marital status	1.377646
Education level	1.160898
Working age	2.875903
Weekly working hours	1.018312
Work shift	1.089348
Income per month	1.018021
Self-rated sleep quality	1.240624
Lack of energy last month	1.249377
Drinking frequency	1.019771
Smokers in 5 best friends	1.039912
Cognition of smoking hazard	1.014899
Self-rated health quality	1.169023
Systolic blood pressure	1.51781
Diastolic blood pressure	1.576722
Heart rate	1.052388
Fasting blood glucose	1.090643
Triglyceride	1.356042
Total cholesterol	2.413103
High-density lipoprotein	1.312888
Low-density lipoprotein	2.163508
Hemoglobin	1.537169
Red blood cell	1.519609
White blood cell	1.127356
Platelet	1.103089
Alanine aminotransferase	1.66562
Aspartate aminotransferase	1.478812

γ-glutamyl transferase	1.281952
Total bilirubin	1.451438
Direct bilirubin	1.467096
Total protein	1.176061
Albumin	1.17137
Urea nitrogen	1.054667
Uric acid	1.103105
Creatinine	1.088525
Alpha-fetoprotein	1.033856
Carcinoembryonic antigen	1.02336
Urine pH	1.032737

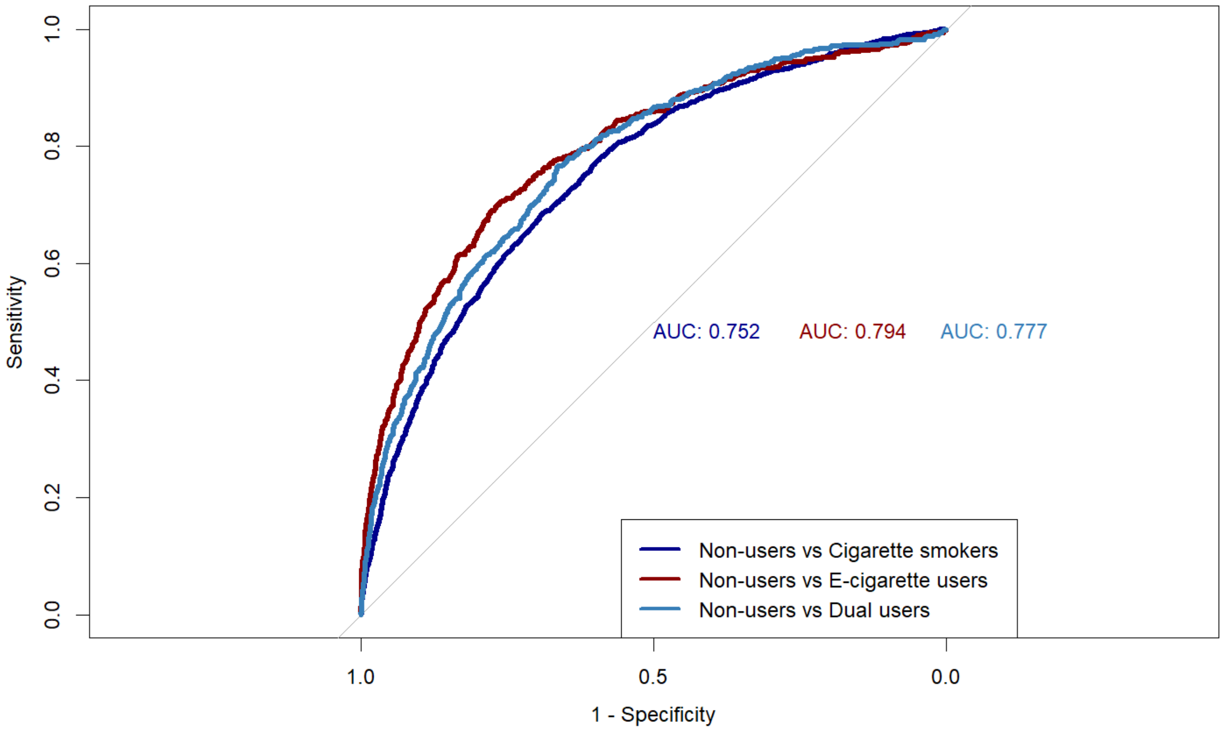

Figure S1 ROC curves of the multinomial logistic regression analysis

Table S2 Multinomial logistic regression results with cigarette smokers and e-cigarette users as references

	Cigarette smokers as a reference				E-cigarette users as a reference	
	E-cigarette users		Dual users		Dual users	
**Characteristics**	**OR (95%CI)**	***P***	**OR (95%CI)**	***P***	**OR (95%CI)**	***P***
Age	1.02 (0.98, 1.06)	0.274	1.05 (1.01, 1.10)	**0.013**	1.03 (0.98, 1.08)	0.208
BMI	0.97 (0.93, 1.01)	0.123	1.02 (0.98, 1.06)	0.315	1.05 (1.00, 1.10)	**0.041**
Marital status: Married	0.59 (0.44, 0.78)	**<0.001**	0.57 (0.44, 0.75)	**<0.001**	0.97 (0.69, 1.38)	0.881
Education level	0.44 (0.34, 0.56)	**<0.001**	0.94 (0.72, 1.23)	0.676	2.15 (1.56, 2.95)	**<0.001**
Working age	0.97 (0.94, 1.00)	0.0678	0.97 (0.94, 1.00)	0.074	1.00 (0.96, 1.04)	0.927
Weekly working hours	0.99 (0.98, 1.00)	0.169	1.00 (0.99, 1.01)	0.727	1.00 (0.99, 1.02)	0.411
Work shift	0.98 (0.89, 1.08)	0.657	1.04 (0.94, 1.15)	0.418	1.06 (0.94, 1.20)	0.317
Income per month	1.02 (0.95, 1.10)	0.577	1.05 (0.97, 1.13)	0.195	1.03 (0.94, 1.13)	0.563
Self-rated sleep quality	0.84 (0.72, 0.98)	**0.023**	0.95 (0.82, 1.11)	0.523	1.13 (0.94, 1.37)	0.189
Lack of energy last month	0.96 (0.85, 1.09)	0.522	1.05 (0.93, 1.19)	0.400	1.10 (0.94, 1.27)	0.236
Drinking frequency: Occasional	0.56 (0.44, 0.71)	**<0.001**	0.80 (0.63, 1.01)	0.059	1.42 (1.06, 1.92)	**0.019**
Regular	0.97 (0.74, 1.27)	0.814	0.89 (0.67, 1.18)	0.430	0.92 (0.66, 1.30)	0.642
Quit	1.56 (0.70, 3.49)	0.279	0.77 (0.28, 2.12)	0.613	0.49 (0.16, 1.52)	0.219
Smokers in 5 best friends	1.03 (0.96, 1.11)	0.443	1.25 (1.16, 1.35)	**<0.001**	1.21 (1.10, 1.33)	**<0.001**
Cognition of smoking hazard	0.77 (0.71, 0.84)	**<0.001**	1.01 (0.93, 1.10)	0.813	1.31 (1.18, 1.45)	**<0.001**
Self-rated health quality	1.27 (1.13, 1.44)	**<0.001**	1.02 (0.91, 1.16)	0.709	0.80 (0.69, 0.93)	**0.0047**
Systolic blood pressure	1.18 (1.06, 1.31)	**0.002**	1.01 (0.91, 1.12)	0.854	0.86 (0.75, 0.98)	**0.023**
Diastolic blood pressure	0.98 (0.85, 1.13)	0.765	0.91 (0.79, 1.05)	0.180	0.93 (0.78, 1.11)	0.40
Heart rate	1.18 (1.06, 1.33)	**0.004**	0.96 (0.85, 1.08)	0.481	0.81 (0.70, 0.94)	**0.005**
Fasting blood glucose	1.07 (0.87, 1.30)	0.518	1.02 (0.83, 1.25)	0.833	0.96 (0.74, 1.23)	0.731
Triglyceride	0.89 (0.74, 1.06)	0.200	0.91 (0.76, 1.08)	0.274	1.02 (0.81, 1.27)	0.873
Total cholesterol	1.19 (0.90, 1.57)	0.223	1.17 (0.88, 1.57)	0.282	0.99 (0.69, 1.41)	0.942
High-density lipoprotein	0.76 (0.45, 1.30)	0.318	0.68 (0.40, 1.18)	0.169	0.90 (0.46, 1.76)	0.753
Low-density lipoprotein	0.80 (0.59, 1.09)	0.161	0.95 (0.69, 1.30)	0.735	1.18 (0.80, 1.73)	0.41
Hemoglobin	1.22 (1.05, 1.42)	**0.010**	1.15 (0.99, 1.34)	0.070	0.94 (0.78, 1.14)	0.53
Red blood cell	0.85 (0.57, 1.28)	0.445	0.63 (0.42, 0.95)	**0.028**	0.74 (0.45, 1.22)	0.239
White blood cell	1.05 (0.97, 1.13)	0.239	0.95 (0.88, 1.03)	0.252	0.91 (0.83, 1.01)	0.064
Platelet	1.02 (0.84, 1.23)	0.846	1.06 (0.88, 1.29)	0.52	1.04 (0.82, 1.33)	0.721
Alanine aminotransferase	1.01 (0.91, 1.14)	0.80	0.99 (0.88, 1.11)	0.811	0.97 (0.84, 1.12)	0.694
Aspartate aminotransferase	1.23 (1.00, 1.51)	**0.054**	0.98 (0.79, 1.20)	0.817	0.79 (0.61, 1.03)	0.085
γ-glutamyl transferase	1.01 (0.93, 1.09)	0.892	1.02 (0.94, 1.11)	0.588	1.02 (0.92, 1.12)	0.741
Total bilirubin	1.00 (0.98, 1.04)	0.748	1.00 (0.97, 1.03)	0.776	0.99 (0.95, 1.03)	0.628
Direct bilirubin	1.04 (0.95, 1.14)	0.355	1.06 (0.96, 1.16)	0.258	1.01 (0.90, 1.13)	0.854
Total protein	1.00 (0.97, 1.03)	0.93	1.00 (0.97, 1.03)	0.833	1.00 (0.97, 1.04)	0.811
Albumin	0.95 (0.90, 0.99)	**0.016**	0.97 (0.92, 1.01)	0.12	1.02 (0.96, 1.08)	0.478
Urea nitrogen	0.96 (0.87, 1.05)	0.375	1.00 (0.91, 1.10)	0.977	1.05 (0.93, 1.18)	0.463
Uric acid	1.06 (0.92, 1.21)	0.421	1.01 (0.88, 1.16)	0.849	0.96 (0.81, 1.14)	0.624
Creatinine	1.02 (0.93, 1.12)	0.666	1.02 (0.93, 1.12)	0.699	1.00 (0.89, 1.12)	0.97
Alpha-fetoprotein	0.91 (0.84, 0.97)	**0.006**	1.00 (0.94, 1.07)	0.91	1.11 (1.02, 1.21)	**0.021**
Carcinoembryonic antigen	1.07 (0.97, 1.19)	0.183	1.00 (0.91, 1.11)	0.933	0.94 (0.83, 1.06)	0.318
Urine pH	0.70 (0.56, 0.88)	**0.002**	1.14 (0.90, 1.43)	0.282	1.63 (1.23, 2.15)	**0.001**

AOR: adjusted odds ratio. MLNR analysis adjusted for age, body mass index (BMI), education level, monthly income, drinking frequency, self-rated sleep quality, work shift, and marital status.

Bold: $p<0.05$ is significant.

OR: odds ratio, 95% CI: confidence interval

Table S3 The effects of accumulated smoking years in the three smoking subgroups

Health indicators	Cigarette smokers		E-cigarette users		Dual users	
	**β(95%CI)**	***P***	**β(95%CI)**	***P***	**β(95%CI)**	***P***
BMI	-0.0104 (-0.0326, 0.0117)	0.356	-0.0109 (-0.0437, 0.0219)	0.516	0.0105 (-0.0279, 0.0490)	0.592
Systolic pressure	-0.1281 (-0.2000, -0.0562)	**<0.001**	-0.0424 (-0.1489, 0.0642)	0.435	-0.1193 (-0.2441, 0.0055)	0.061
Diastolic pressure	0.0538 (-0.0008, 0.1083)	0.053	0.0771 (-0.0036, 0.1578)	0.061	-0.0460 (-0.1406, 0.0487)	0.341
Heart rate	-0.0147 (-0.0864, 0.0570)	0.688	0.0906 (-0.0154, 0.1967)	0.094	-0.0425 (-0.1668, 0.0819)	0.503
Fasting blood glucose	-0.0053 (-0.0094, -0.0012)	**0.011**	-0.006 (-0.0121, 0.0001)	0.052	-0.0079 (-0.0150, -0.0008)	**0.029**
Triglyceride	-0.0003 (-0.005, 0.0044)	0.902	-0.0057 (-0.0127, 0.0013)	0.108	-0.0064 (-0.0146, 0.0018)	0.125
Total cholesterol	0.0018 (-0.0011, 0.0047)	0.227	0.0075 (0.0032, 0.0117)	**0.001**	0.0033 (-0.0018, 0.0083)	0.203
High-density lipoprotein	-0.0019 (-0.0034, -0.0003)	**0.017**	-0.0053 (-0.0076, -0.0030)	**<0.001**	-0.0039 (-0.0066, -0.0013)	**0.004**
Low-density lipoprotein	0.0004 (-0.0024, 0.0031)	0.799	-0.0049 (-0.0089, -0.0009)	**0.017**	-0.0015 (-0.0062, 0.0032)	0.539
Hemoglobin	0.0587 (0.0099, 0.1076)	**0.018**	0.1018 (0.0296, 0.1740)	**0.006**	0.1212 (0.0366, 0.2058)	**0.005**
Red blood cell	-0.0031 (-0.005, -0.0013)	**0.001**	-0.0039 (-0.0066, -0.0011)	**0.006**	-0.0054 (-0.0086, -0.0022)	**0.001**
White blood cell	0.0278 (0.0174, 0.0382)	**<0.001**	0.0304 (0.0149, 0.0458)	**<0.001**	0.0179 (-0.0002, 0.0360)	0.053
Platelet	0.1098 (-0.2543, 0.4739)	0.554	0.0637 (-0.4749, 0.6024)	0.817	0.2922 (-0.3391, 0.9235)	0.364
Alanine aminotransferase	0.0244 (-0.0390, 0.0878)	0.451	0.014 (-0.0798, 0.1079)	0.769	-0.0243 (-0.1343, 0.0857)	0.665
Aspartate aminotransferase	-0.0502 (-0.0804, -0.0200)	**0.001**	0.0179 (-0.027, 0.0627)	0.435	-0.0282 (-0.0807, 0.0243)	0.293
γ-glutamyl transferase	0.0551 (-0.0427, 0.1528)	0.269	0.1227 (-0.0218, 0.2673)	0.096	0.1272 (-0.0423, 0.2966)	0.141
Total bilirubin	-0.017 (-0.0447, 0.0107)	0.229	-0.0348 (-0.0758, 0.0062)	0.096	-0.0261 (-0.0742, 0.0220)	0.287
Direct bilirubin	-0.0043 (-0.0133, 0.0046)	0.345	0.0076 (-0.0056, 0.0209)	0.258	0.0006 (-0.0150, 0.0161)	0.942
Total protein	-0.0436 (-0.0709, -0.0164)	**0.002**	-0.0797 (-0.1200, -0.0395)	**<0.001**	-0.0295 (-0.0768, 0.0178)	0.221
Albumin	0.0067 (-0.0112, 0.0245)	0.463	-0.0088 (-0.0352, 0.0175)	0.511	-0.0169 (-0.0478, 0.0141)	0.285
Urea nitrogen	0.0072 (-0.0016, 0.016)	0.106	0.0092 (-0.0039, 0.0222)	0.168	0.0015 (-0.0138, 0.0167)	0.849
Uric acid	-0.121 (-0.6832, 0.4412)	0.673	0.1256 (-0.7062, 0.9574)	0.767	0.2937 (-0.6812, 1.2686)	0.555
Creatinine	-0.0244 (-0.1061, 0.0573)	0.559	-0.0899 (-0.2108, 0.0309)	0.145	0.0265 (-0.1152, 0.1681)	0.714
Alpha-fetoprotein	0.0217 (0.0098, 0.0335)	**<0.001**	-0.003 (-0.0206, 0.0146)	0.737	0.0240 (0.0034, 0.0446)	**0.022**
Carcinoembryonic antigen	0.0056 (-0.0025, 0.0137)	0.174	0.0212 (0.0092, 0.0332)	**0.001**	0.0109 (-0.0032, 0.0249)	0.130
Urine pH	0.0029 (-0.0006 0.0063)	0.103	0.0001 (-0.005, 0.0052)	0.980	0.0061 (0.0001, 0.0120)	**0.046**

Bold: p<0.05 is significant.

Table S4 GVIF values for the multiple linear regression analysis

Variables	GVIF
Age	2.686239
Marital status	1.299388
Education level	1.162788
Working age	2.585523
Weekly working hours	1.026122
Work shift	1.085288
Income per month	1.023988
Self-rated sleep quality	1.222010
Lack of energy last month	1.224724
Drinking frequency	1.030056
Smokers in 5 best friends	1.065766
Cognition of smoking hazard	1.017952
Self-rated health quality	1.147149
Systolic blood pressure	1.503574
Diastolic blood pressure	1.568409
Heart rate	1.051065
Fasting blood glucose	1.104845
Triglyceride	1.364499
Total cholesterol	2.295366
High-density lipoprotein	1.308371
Low-density lipoprotein	2.051424
Hemoglobin	1.533625
Red blood cell	1.515775
White blood cell	1.143653
Platelet	1.104655
Alanine aminotransferase	1.641110
Aspartate aminotransferase	1.488153
γ-glutamyl transferase	1.289997
Total bilirubin	1.440944
Direct bilirubin	1.457369
Total protein	1.158738
Albumin	1.159436
Urea nitrogen	1.051666
Uric acid	1.086714
Creatinine	1.093751
Alpha-fetoprotein	1.043419
Carcinoembryonic antigen	1.028781
Urine pH	1.037622
Accumulated smoking years: Three smoking subgroups	1.047192

© 2025 Xiao L. et al.
